# Supplementary material for: The oxytocin receptor gene polymorphism rs2268491 and serum oxytocin alterations are indicative of autism spectrum disorder: A case-control paediatric study in Iraq with personalized medicine implications
Source: PLoS One. 2022 Mar 22;17(3):e0265217. doi: 10.1371/journal.pone.0265217 (PMC8939799; doi:10.1371/journal.pone.0265217)
Supplement: S1 Table — (DOCX) [file pone.0265217.s002.docx]

**Supplementary Table S1**. Instruments and equipment used in the study

| **No.** | **Instruments and equipment** | **Manufacturers/ country** |
| --- | --- | --- |
| 1 | Autoclave supply | OLMAR Labs, Gijon City, Spain |
| 2 | Anticoagulant vacuum tube | Jordan Biolabs, Amman City, Jordan |
| 3 | Centrifuge | Memmert GmbH Co., Schwabach, Germany |
| 4 | Deep freezer | AHT, GmbH CO., |
| 5 | Distillatory | Lab Unlimited, Camberley, Surrey, UK |
| 6 | Digital camera | Sony, Minato City, Tokyo, Japan |
| 7 | Electrophoresis power | ThermoFisher Scientific, Waltham, Massachusetts, USA |
| 8 | Microcentrifuge, High Speed Cooling centrifuge | Hettich Lab, Germany |
| 9 | Eppendorf tubes (1.5mL, 0.5mL) | Eppendorf Co., Hamburg, Germany |
| 10 | Gel documentation system (Vision) | Thomas Scientific LLC Co., Swedesbora, USA |
| 11 | Microscope | Olympus Co., Shinjuku, Tokyo, Japan |
| 12 | Microscope slides | Boenmed Ltd, Co., Jiangsu, China |
| 13 | Magnetic Stirrer with hotplate | Stuart, Paris, France |
| 14 | Micropipette tips (10μL, 100μL, 200μL, 1000μL) | **Suzhou ACE Biomedical Technology Co., Ltd.** Jiangsu, China |
| 15 | Nanodrop spectrophotometer | ThermoFisher Scientific, Waltham, Massachusetts, USA |
| 16 | Sensitive Balance | Sartorius/Germany |
| 17 | Thermocycler PCR | Biometra, Goettingen, Germany |
| 18 | UV Transilluminator | Cleaver, Wawickshire, UK |
| 19 | Water Bath | **Ningbo Scientz Biotechnology Co., Ltd.** Zhejiang, China |
