# Supplementary material for: The oxytocin receptor gene polymorphism rs2268491 and serum oxytocin alterations are indicative of autism spectrum disorder: A case-control paediatric study in Iraq with personalized medicine implications
Source: PLoS One. 2022 Mar 22;17(3):e0265217. doi: 10.1371/journal.pone.0265217 (PMC8939799; doi:10.1371/journal.pone.0265217)
Supplement: S2 Table — (DOCX) [file pone.0265217.s003.docx]

**Supplementary Table S2**. Chemicals and reagents for DNA extraction and polymerase chain reaction.

| **No.** | **Name** | **Source** |
| --- | --- | --- |
| **1** | Pure Blood Genomic DNA Mini Kit (Column Extraction Kit), Components: | Anatolia, Istanbul, Turkey |
|  | Proteinase K |  |
|  | PBS solution |  |
|  | Buffer CL |  |
|  | CW1 solution (concentrate) |  |
|  | CW2 solution (concentrate) |  |
|  | Elution buffer (CE) |  |
|  | Buffer TBP |  |
|  | Collection tube 2ml |  |
| 2 | 100bp-3000bp DNA ladder | ABM Research Ltd., Co., Toronto, ON, Canada |
| 3 | Master Mix | ABM Research Ltd., Co., Toronto, ON, Canada |
| 4 | Nuclease free water | ABM Research Ltd., Co., Toronto, ON, Canada |
| 5 | Primers | IDT, Newark, NJ, USA |
| 6 | Bromophenol blue dye | ABM Research Ltd., Co., Toronto, ON, Canada |
| 7 | Ethidium bromide | ABM Research Ltd., Co., Toronto, ON, Canada |
