# Supplementary material for: The oxytocin receptor gene polymorphism rs2268491 and serum oxytocin alterations are indicative of autism spectrum disorder: A case-control paediatric study in Iraq with personalized medicine implications
Source: PLoS One. 2022 Mar 22;17(3):e0265217. doi: 10.1371/journal.pone.0265217 (PMC8939799; doi:10.1371/journal.pone.0265217)
Supplement: S3 Table — (DOCX) [file pone.0265217.s004.docx]

**Supplementary Table S3**. Standard Lab Protocols

| **Protocol Step** | **Protocol: Genomic DNA Extraction** **using the** **Column-pure blood Genomic DNA Mini Kit, Applied Biological Materials (Anatolia Turkey)** |
| --- | --- |
| 1 | Add 200μl of blood into a 2ml centrifuge tube, followed by 2 volumes of buffer TBP, vortex gently, leave for 1 minute until complete lysis occurs |
| 2 | Centrifuge tubes at 800 rpm for 1 minute, carefully discard the supernatant, then wash the precipitate twice with 500μl TE buffer twice, centrifuging at 8000 rpm for 1minute during each wash. The final precipitate appears white |
| 3 | Add 20μl of proteinase K (10mg/mL) to the precipitate and mix well. Add 200μl of buffer CL, vortex, incubate at 56°C for 10 minutes |
| 4 | 200μl ethanol (96–100%) was added to each sample and pulse-vortexed for 15 seconds |
| 5 | The sample mixture was added to the spin column (binding step) and centrifuged at 11,000 rpm for 10 minutes. The flow-through was discarded together with the collection tube and the spin column placed into a new microcentrifuge tube. |
| 6 | The washing steps involved 500μl of W1 solution and microcentrifugation at 10000 rpm for 1 minute, followed by 500 µl volume of buffer W2 for a further 1 minute. The flow-through together with the collection tube was discarded and the spin column was placed in a new microcentrifuge tube |
| 7 | The empty spin column was centrifuged for 1 minute at 12,500 rpm. The spin column was placed in a new microcentrifuge tube and a 2-minute interval permitted evaporation of residual ethanol. |
| 8 | A 50µl volume of elution buffer was added to the centre of the spin column followed by a 5-minute incubation step. The spin column was centrifuged for 12,500 rpm for 1 minute in a microcentrifuge. |
| 9 | The liquid flow-through containing the genomic DNA isolated was stored at -20°C |
| **Protocol step** | **Protocol: Preparation of 1% agarose gels to check quality of extracted DNA from patient’s blood samples using electrophoresis** |
| 1 | 1.0 g agarose was added to 100 mL of TAE buffer (1X) and microwave melted. Each DNA sample was mixed with 1X gel loading dye prior to loading into the gel. |
| 2 | A final concentration of 0.5 µg/mL ethidium bromide was added to the melted agarose. |
| 3 | The liquid agarose was poured into the casting tray of the submarine gel electrophoresis unit (Cleaver Scientific Co., UK). |
| 4 | After gel solidification, the casting tray was immersed in the submarine tank. TAE buffer (1X) was added to completely submerge the gel. |
| 5 | Each DNA sample was mixed with 1X gel loading dye prior to loading into the gel. The electrophoresis process was conducted at 5-8 voltage/cm for 45 minutes |
| **Protocol step** | **Protocol: Preparation of 2% agarose gels for electrophoresis analysis of PCR products** |
| 1 | Add 2 g agarose and 40ml of 1X TBE, completing the volume to 100ml distal water and heat at 100°C, leave to cool (50°C). |
| 2 | 5µl of ethidium bromide stain was added into agarose gel solution |
| 3 | Agarose gel solution was poured into the casting tray, the comb was added, and left to solidify for 15 minutes at room temperature, after that the comb was removed gently from the tray to create neat gel wells |
| 4 | 10µl of PCR product was added into each gel well in gel and 5µl of (100bp ladder) in one well. |
| 5 | The gel tray was fixed in electrophoresis chamber and filled gently with 1X TBE buffer. Then electric current was performed at 80- 100 volt and 80 mA for 1hour. |
| 6 | PCR products were visualized by using UV transilluminator |
