# Supplementary material for: The oxytocin receptor gene polymorphism rs2268491 and serum oxytocin alterations are indicative of autism spectrum disorder: A case-control paediatric study in Iraq with personalized medicine implications
Source: PLoS One. 2022 Mar 22;17(3):e0265217. doi: 10.1371/journal.pone.0265217 (PMC8939799; doi:10.1371/journal.pone.0265217)
Supplement: S4 Table — (DOCX) [file pone.0265217.s005.docx]

**S4 Table:** The sequences of allele specific primers of oxytocin receptor diallelic gene rs2268491 (C/T) and PCR information

| **Oxytocin SNP rs2268491 (C/T): Alleles T & C Primer Sequences** | **PCR recipe and cycle conditions** |
| --- | --- |
| - **T Allele:**   Forward primer sequence: 5’- GTCTGGGTG AAGAGCCAGAT -3’  Reverse primer sequence: 5’- GAGTTTGCAGTTTTGACAAGGAT -3’  T Allele Base pair -product size: 342 bp   - **A Allele:**   Forward primer sequence: 5’- GTCTGGGTG AAGAGCCAGA**C** -3’  Reverse primer sequence: 5’- GAGTTTGCAGTTTTGACAAGGAT -3’  A Allele Base pair -product size: 342 bp   - **Control reactions (**583 bp)**:**   Forward,  5-CAAGGCCTCC CCAGAGTACC-3  Reverse- 5 GAGTTTGCAGTTTTGACAAGGAT-3 | *Recipe volumes:*   - Genomic DNA: 3.0 µL - Forward-primer: 2.0 µL - Reverse-primer: 2.0 µL - 2XPCR master mix: 12.5 µL - Nuclease Free Water: 5.5 µL - Total PCR-reaction volume: **25 µL**   *Conditions:*   - 1 cycle: Initial denaturation: 95 ^o^C, 5 min - 30 cycles, each cycle:   - Denaturation: 94 ^o^C, 1 min   - Annealing: 59 ^o^C, 1 min   - Extension: 72 ^o^C, 25 seconds - 1cycle: Final extension: 72 ^o^C, 10 min |
