# Supplementary material for: The oxytocin receptor gene polymorphism rs2268491 and serum oxytocin alterations are indicative of autism spectrum disorder: A case-control paediatric study in Iraq with personalized medicine implications
Source: PLoS One. 2022 Mar 22;17(3):e0265217. doi: 10.1371/journal.pone.0265217 (PMC8939799; doi:10.1371/journal.pone.0265217)
Supplement: S5 Table — (DOCX) [file pone.0265217.s006.docx]

**S5 Table:** OXT levels of control and autistic groups with related and unrelated parents.

| Group | N | Related parent | | Unrelated parent | |
| --- | --- | --- | --- | --- | --- |
|  |  | N | Mean± SD | N | Mean± SD |
| Control | 60 | 22 (36.66) | 76.83±13.08 | 38 (63.33) | 73.21±11.58 |
| ASD | 60 | 22 (36.66%) | 151.83±6.64 | 38 (63.33%) | 146.19±9.31 |
| Mild ASD | 39 | 14 (35.89%) | 161.72±6.12 | 25 (64.1%) | 168.33±4.35 |
| Moderate ASD | 13 | 4 (30.76%) | 163.56±7.12 | 9 (69.23%) | 141.85±4.68 |
| Severe ASD | 8 | 4 (50%) | 130.06±5.02 | 4 (50%) | 128.1±7.21 |

S5 Table shows that there are no significant differences in OXT levels between related and unrelated parental groups (p>0.05).
