# Supplementary material for: The oxytocin receptor gene polymorphism rs2268491 and serum oxytocin alterations are indicative of autism spectrum disorder: A case-control paediatric study in Iraq with personalized medicine implications
Source: PLoS One. 2022 Mar 22;17(3):e0265217. doi: 10.1371/journal.pone.0265217 (PMC8939799; doi:10.1371/journal.pone.0265217)
Supplement: Attachment — Submitted filename: answer to reviewers.docx [file pone.0265217.s008.docx]

**Response to Reviewers**

1. **Criterion 3:**
a. (1) ***Authors should clarify sampling method and justify sample size.***

Answer: (lines 126 paragraph manuscript) literature searches were performed to assess the prevalence of autism spectrum disorder. An estimated 1 % of the world’s population has autism spectrum disorder, with recent figures of 1.5%. Since the prevalence of the disease is a relatively uncommon event at 1 out of 100 cases, a case control study was selected as the most efficient sampling method to investigate autism among the Iraqi Arab population. A study sample size of N=120 (60 ASD and 60 controls) was feasible to manage in the face of opposition to the study from local tribal and religious restrictions. Our study had a 3:1 male to female ratio in the ASD and control populations. The DSM-5 states that “autism spectrum disorder is diagnosed four times more often in males than in females.”(American Psychiatric Association 2013), and most recent research suggest that it is closer to 3:1 (Loomes, Hull, and Mandy 2017). Therefore the study population is reflective of the gender ratio in ASD and provides a useful model to explore specific genetic associations.

b. ***(2) Define severity of ASD.***

Answer: This was delineated in the PLOS PDF lines 145-182 as follow:

The severity was defined according to the Childhood Autism Rating Scale as follow:

The patients with ASD were stratified according to severity into the following sub-groups (20): mild ASD (n=39), moderate ASD (n=13) and severe ASD (n=8) according to the Childhood Autism Rating Scale
(CARS) (21). The CARS is a diagnostic assessment method that rated individuals on a scale ranging from normal to severe and yields a composite score ranging from non-autistic to mildly autistic, moderately autistic, or severely autistic. The scale was used to observe and subjectively rate the following fifteen items in both ASD and case control subjects. Each of the following 15-items were scored in patients according to seven levels of severity by interview:
1. Relationship to people
2. Imitation
3. Emotional response
4. Body
5. Object use
6. Adaptation to change
7. Visual response
8. Listening response
9. Taste-smell-touch response and use
10. Fear and nervousness
11. Verbal communication
12. Non-verbal communication
13. Activity level
14. Level and consistency of intellectual response
15. General impressions

c. ***(3) Provide required details for all used kits and reagents, such as manufacturer, city, and country.***

Answer: done. These were delineated in supplementary Table S1, S2

**S1 Table**. Instruments and Equipment used in the study.

**S2 Table**. Chemicals and reagents for DNA extraction and polymerase chain reaction.

d. ***(4) Experimental details on Isolation of genomic DNA and Agarose gel electrophoresis are commonly used techniques so that they should be summarized in the main text and the technical details can be better provided as supplementary.***

Answer:

The experimental details for both isolation of genomic DNA and preparation of gels for electrophoresis are now summarized in the main manuscript with full lab protocols described in supplementary table 3.

e. ***(5) Did the authors test data for normality?***

Answers: The Kolmogorov-Smirnov test was used to test normality, which showed a normal distribution, methods (statistical analysis): paragraph 270. The test for all variables was greater than 0.05 so that samples are normally distributed (Table 2).

f. Did the author consider confounders “e.g., the Median age of control is lower than that of ASD, which may affect serum OXT level”.

Answer. Ages were matched between the ASD and control group. There were no significant differences in age distribution between studied and control group P=0.708 (Table 2).

g. (7) In table 2, authors should describe which statistical tests were used; moreover, data are presented as mean (SD) but some appear to be not normally distributed.

Answer: done. Analysis of Variance (ANOVA) test were used for statistical analysis as the data were normally distributed which was ensured by doing Kolmogorov-Smirnov test (Table 2).

h. (8) In addition “The ANOVA test found statistically significant difference (p=0.032) in OXT levels according to severity of ASD, as shown in Table 2”. Please, specify the difference?

Answer: done. The oxytocin level was 165.02±28.03 pg/mL in mild cases, 152.78±27.86 pg/mL in moderate cases, and 129.09±11.04 pg/mL in severe cases. Furthermore, there were significant differences in oxytocin level when comparing mild and severe cases (p=0.001), moderate and severe cases (p=0.034). On other hand there were no significant differences in oxytocin level between mild and moderate cases (p=0.178) (Table 2, table footnotes).

i. (9) In table 2, it seems that Oxytocin levels are lower in severe ASD (higher in mild ASD), which may be associated with older age.

Answer. Done. Oxytocin level was higher in mild cases (165.02±28.03 pg/mL) than severe cases (129.09±11.04 pg/mL). However there was no significant correlation between age of the patient and oxytocin level as stated in the manuscript in lines 554 and Supplementary Figure 1 (P= 0.396, R=-0.140, R2=0.0196; Supplementary Fig 1).

j. (10) It is interesting to see that healthy controls had highest OXT levels in the CC genotype, while ASD subjects had the highest OXT in the TT genotype; it would be meaningful to explain/discuss such findings.

Answer. Done (Discussion, main manuscript). In stratified ASD patients, serum oxytocin levels were higher in the mild category with the polymorphism genotype TT. This suggests that patients with milder ASD symptoms and the TT genotype may have evolved an ability to upregulate OXT levels to help or try to compensate for the cerebral social response skills deficiency caused by the OXTR polymorphism. The higher amounts of OXT will help to improve facial processing and human interpersonal contact. As showed in previous study D LoParo et al 2015 showed oxytocin receptor gene rs2268491 (‘T’ allele is risk-inducing) in the meta-analysis, these SNPs were shown to be substantially related with ASD, implying that signals from these SNPs may represent a shared connection with ASD. Liu x, et al. 2010 showed two SNPs (rs2268491 and rs2254298), which were significantly associated with ASD in there study in which re 2268491 with allele C predominant.

2. Criterion 4. “Conclusions are presented in an appropriate fashion and are supported by the data.” Authors have to revise the conclusion of their study to be based on study findings (e.g., “The measurement of peripheral OXT levels and OXTR genetic alterations provide a simple dual-biomarker test to gauge social functioning in the ASD patient for diagnostic, monitoring and support purposes”; however, this study didn’t investigate monitoring and support aspects). Other suggested implications/recommendations based on study findings/conclusion should be separated from the conclusion itself.

**Answer: Done.** The final conclusion paragraph has been amended, the implications/recommendations of the study have been presented as such from lines 485

3. Criterion 5. “The article is presented in an intelligible fashion and is written in standard English.”
a. (1) Table 1: better to show ASD and controls in columns and variables as rows. Please, provide other important data, such as gender and comorbid conditions.

Answer. Done. Table 1 was corrected as requested by the reviewers.

b. (2) The article contain several errors related to English language, spelling, and grammar.  *PLOS ONE* does not copyedit accepted manuscripts, so the language in submitted articles must be clear, correct, and unambiguous. We may reject papers that do not meet these standards. If the language of a paper is difficult to understand or includes many errors, we may recommend that authors seek independent editorial help before submitting a revision.

Done.

4. Criterion 6. “The research meets all applicable standards for the ethics of experimentation and research integrity.”. The authors repeat the ethics statement twice, once under study populations “Formal parental consent was obtained from families who expressed interest in the study, who were provided with consent sheets and a questionnaire to complete” and under Ethical approval “All patients were informed about the purpose of the work, and a written consent obtained from each patient.”. Authors should provide this information once. Moreover, did they obtain consent from the patients “children?”, parents, or both?

Answer: In fact, written consent was taken from the father of the ASD child only who is in charge and responsible for any matter regarding the ASD patients according to the societal customs in Iraq. The ethics statement has been updated as recommended. Please refer to paragraph lines 272

Criterion 7. “The article adheres to appropriate reporting guidelines and community standards for data availability.”

a. (1) In the abstract and discussion, authors seem to selectively report some findings in an inappropriate manner, such as “study characteristics in the ASD population revealed a high level of consanguinity (36.66%)” although the percentage of consanguinity was higher among control group (40%).

Answer: The sample size might be behind this variation which shows that the percentage of consanguinity was higher among control group. Much larger sample sizes will facilitate a clear picture on these variations. The consanguinity findings are now fully reported in the results and discussion sections lines 282 and 403:

- Results: The rate of consanguinity among the Arabic study populations was high at 36.66% and 40% in the ASD and healthy control groups (Table 1).
- Discussion: Given that the consanguinity rate worldwide is 10.4% (7), the rates observed in both the ASD and control study populations are at least three fold higher.

b. (2) The authors stated that “all data are fully available without restriction” but they did not provide unidentified patients’ data. Authors are required to make all data underlying the findings described fully available, without restriction, and from the time of publication. PLOS allows rare exceptions to address legal and ethical concerns, but this has to be completely explained. Please, review PLOS Data Policy.

Done. All data is available as supplementary material as Supplementary S1 Appendix.

5. Data sharing statement.

Answer: All the data cannot be made publically available as a results of local institution policy. However, it can be made available on a reasonable request from the corresponding author or from the department of pathology and forensic medicine (third party).

Contact information of the department of pathology and forensic medicine is: Email: [pathology.med@uokufa.edu.iq](mailto:pathology.med@uokufa.edu.iq)

(email included in the cover letter)

6. Please amend either the title on the online submission form (via Edit Submission) or the title in the manuscript so that they are identical.

Amended

7. PLOS PDF (phrase data not shown).

Done. This phrase has been removed, and the OXT and patient age Supplementary Fig 1 is now included.
