# Supplementary material for: The oxytocin receptor gene polymorphism rs2268491 and serum oxytocin alterations are indicative of autism spectrum disorder: A case-control paediatric study in Iraq with personalized medicine implications
Source: PLoS One. 2022 Mar 22;17(3):e0265217. doi: 10.1371/journal.pone.0265217 (PMC8939799; doi:10.1371/journal.pone.0265217)
Supplement: Attachment — Submitted filename: response to reviewers 070222.docx [file pone.0265217.s009.docx]

**Response to Reviewers**

**PLOS Editor specific revisions:**

The authors would like to thank the PLOS editor for these helpful manuscript suggestions.

- **Previous comment: Authors should clarify sampling method and justify sample size**.

**New comment to address**: Please, provide calculation of the sample size that would be required for this study. If the number enrolled in this study satisfy the calculated sample size, this would be perfect. If not, you can acknowledge this as one of the study limitations and provide justifications, including feasibility issues.

**Response from authors:**

Methods, Lines (131-144) A sample size of N=36 was calculated as the minimal requirement to determine serum oxytocin alterations in the study cohort. The study variable of interest (peripheral oxytocin alterations) was identified as dichotomous (proportion). The sample size was calculated using the equation: N = 4 * Zα2 * p (1 − p)/w2. Where Zα is the confidence level, W is the width of the confidence interval and P is the study estimate of the proportion to be measured. The proportion of ASD patients who present with oxytocin alterations compared to controls was estimated at about 90% (S1 Appendix) with a selected confidence interval of ± 10 [16]

N = 4 * 1.96 * 0.9 (1 − 0.9)/0.22 = 17.64 (18 cases of ASD)

Minimum sample size for 1:1 case-control study cohort: N=36

Please note that a bigger sample size is ideal for estimating a single OXT SNP in a population (about N=250), this is discussed in the new study limitation section of the discussion (lines 508 paragraph).

- **Previous comment**: Did the author consider confounders “e.g., the Median age of control is lower than that of ASD, which may affect serum OXT level”.

**New comment to address:** Thank you for your important clarification. However, as shown in Table 2, the mean age for controls is lower than that of patients, and the p-value may become significant if larger number was included. This underscores the importance of sample size calculation

**Responses from authors:** Thank you for raising the issue of confounders. The study is of a case control age and gender matched design which should reduce effects of these confounder variables. Although the mean age of patients with ASD is slightly higher - it is not significant (Table 1). The sample size calculations indicates that the study numbers are sufficient to investigate the variable of serum oxytocin.

- **Previous comment:** It is interesting to see that healthy controls had highest OXT levels in the CC genotype, while ASD subjects had the highest OXT in the TT genotype; it would be meaningful to explain/discuss such findings.

**New comment:** Thank you for these important discussions/thoughts. However, authors are encouraged to expand the discussion of this point in the main text (please, integrate the rest of your response, including the references, in the main text).

**Responses from authors:**  The discussion of this key finding of the how OXT levels can differ in relation to OXTR genotype has been reworked and expanded in relation to social functioning with references (lines 465 to 488)

- New comment: Thank you for your important clarifications. Please, integrate your comments regarding sample size in the main text.

**Response:** Done. Refer to the study limitation section of the discussion (lines 508 paragraph).

- Please, provide the age of study participants in table 1 (with other characteristics) rather than table 2.

**Response:** Done

- It is not necessary to provide the p-value for the Kolmogorov-Smirnov test in table 2. Instead, just report this in the table footnote or results section.

**Response:** Done

- Authors are encouraged to acknowledge and discuss study limitations and generalizability of findings.

**Response:** done. (Lines 507-519) ‘Ideally the conclusions presented in this study should be generalizable to the wider Iraqi Arab population. Therefore the study samples must be representative of the population and adequate in number (33). Feasibility issues in the Middle and South Euphrates population region limited recruitment of large sample sizes in the current study. Although the estimated study sample size was sufficient for the serum OXT analysis, numbers are still inadequate to generalize to the entire population. For example, consanguinity was high among the ASD populations as expected, but also the control group. The sample size might explain why the consanguinity proportions are higher among control patients. Sample size also affects the association between SNP markers and disease. Testing a single SNP marker in a 1:1 case control study requires 248 cases to achieve 80% statistical power according to the allelic genetic model, under certain assumptions (34). Insufficient study numbers are a limitation of the current investigation of SNP rs2254298.

**Reviewer 2:**

The authors would like to thank reviewer 2 for these helpful revisions.

- Most recent prevalence of ASD should be cited:
  E.g.: Bougeard Clémence, Picarel-Blanchot Françoise, Schmid Ramona, Campbell Rosanne, Buitelaar Jan. Prevalence of Autism Spectrum Disorder and Co-morbidities in Children and Adolescents: A Systematic Literature Review, Frontiers in Psychiatry, 12, 2021 DOI=10.3389/fpsyt.2021.744709

**Response:** This is an excellent suggestion, we have updated our introductory prevalence of ASD literature, citing ‘recent estimates of 1.70 and 1.85% in US children aged 4 and 8 years respectively’ (lines 81-82)

- The significantly high oxytocin in mild ASD patients compared to moderate and severe should be explained and supported by other studies if possible.

**Response:** Done. Please refer to discussion lines 441- 457

- The trend for elevated OXT in this study agrees with other studies (14-16). However, the use of OXT as a biomarker of ASD is not straightforward, as these results are contradictory with those reporting lower OXT in ASD patients (10-12). However, within the stratified ASD population in this study, the highest OXT levels occurred in the mild subgroup, while the lowest OXT levels occurred in the severe group. Our study found that ASD severity categories had statistically significant (p=0.032) difference in OXT levels. This trend does agree with the previous study of ASD children in Iraq (12). Here OXT level were highest in mild autistic patients with a significant decrease (p<0.05) in moderate autistic patients, and a highly significant decrease (p<0.01) in severe autistic patients compared with control. Interestingly, this variability in OXT levels in stratified ASD patients reported both Iraqi studies may relate to a key finding of the Stanford study (27). The US investigators noted that pretreatment blood OXT concentrations also predicted response to intranasal OXT treatment(27). Those individuals with the lowest pretreatment OXT concentrations showed the greatest social improvement (27). Therefore, the ASD severe patients with lowest OXT in our study could be suitable candidates for individualized intranasal therapy with OXT to boost their social functioning. Individuals with autism do not outgrow autism (This sentence is not clear please clarify or delete)

**Response:** Done. Sentence is updated to: ‘Autism is a lifelong neurodevelopmental disorder’.

- “Ignoring early and accurate diagnosis of this disorder might lead to secondary disorders such as depression and anxiety (Reference is needed). Please see the reference below: Hollocks MJ, Lerh JW, Magiati I, Meiser-Stedman R, Brugha TS. Anxiety and depression in adults with autism spectrum disorder: a systematic review and meta-analysis. Psychol Med. 2019 Mar;49(4):559-572. doi: 10.1017/S0033291718002283. Epub 2018 Sep 4. PMID: 30178724.

**Response:** Done. Thank you for the reference suggestion.

- “These results suggest the ROC curve could become the gold standard for the identification of parameters that are sensitive and specific enough to support ASD diagnosis”. ROC curves are already known as excellent statistical tool in the field of biomarkers, so this statement should be corrected.

**Response:** Done. ROC curves are now discussed as an existing ‘gold standard statistical tool (lines 432)

- The authors stated that “within the stratified ASD population in this study, the highest OXT levels occurred in the mild subgroup, while the lowest OXT levels occurred in the severe group. This should be explained and supported at least with the fact that nasal oxytocin is recommended to decrease the severity of ASD symptoms (Support is mandatory).

**Response:** Done. (Lines 451-456). The US investigators noted that pretreatment blood OXT concentrations also predicted response to intranasal OXT treatment (27). Those individuals with the lowest pretreatment OXT concentrations showed the greatest social improvement (27). Therefore, the ASD severe patients with lowest OXT in our study could be suitable candidates for individualized intranasal therapy with OXT to boost their social functioning.

- The significant difference between mild, moderate and severe ASD should be clearly presented in the table not only in the text. Roc curves for mild, moderate and severe should be presented independently

**Response:** Done. Figure 1 has been updated to show all ROC curves independently, including mild, moderate and sever (Fig 1C-E).

- The authors mentioned that “This suggests that patients with milder ASD symptoms and the TT genotype may have evolved an ability to upregulate OXT levels to help or try to compensate for the cerebral social response skills deficiency caused by the OXTR polymorphism. The higher amounts of OXT will help to improve facial processing and human interpersonal contact” (Again support your suggestion).
  Please go through the manuscript below it might help
  https://www.nature.com/articles/s41598-020-79109-0.pdf
  Horiai, M., Otsuka, A., Hidema, S. et al. Targeting oxytocin receptor (Oxtr)-expressing neurons in the lateral septum to restore social novelty in autism spectrum disorder mouse models. Sci Rep 10, 22173 (2020). https://doi.org/10.1038/s41598-020-79109-0
  Gene expression analysis shows that Oxt mRNA is up-regulated in brain and bone which indicate that Oxt is adaptive and important in restoring the homeostasis of the body.

**Response:** Done. Suitable discussion material (lines 484-487) has been added from the suggested paper. ‘A recent mouse ASD model study demonstrated that targeting OXTR-expressing neurons in the lateral septum restores social skills (31). The results of the current study and previous research (27, 31) suggest that OXT is adaptive and can restore the homeostasis of the body.
